# Differences between high‐precision Alzheimer's disease plasma and PET biomarkers among cognitively normal older Black and White adults

**DOI:** 10.1002/alz.092912

**Published:** 2025-01-09

**Authors:** Omonigho M Bubu, Allal Boutajangout, Ludovic Debure, Alok Vedvyas, Anthony Q Briggs, Mark A Bernard, Joshua L Gills, Elena Valkanova, Alfred Mbah, Mobeena Ghuman, Girardin A Jean‐Louis, Rebecca A Betensky, Karyn Marsh, Henry Rusinek, Ricardo S. Osorio, Thomas Wisniewski, Arjun V. Masurkar

**Affiliations:** ^1^ NYU Grossman School of Medicine, New York, NY USA; ^2^ NYU School of Medicine, New York, NY USA; ^3^ University of South Florida, Tampa, FL USA; ^4^ NYU College of Global Public Health, New York, NY USA

## Abstract

**Background:**

We evaluated whether baseline differences existed in various plasma Alzheimer’s disease (AD) biomarkers in cognitively normal participants racialized as Black or White.

**Method:**

This cross‐sectional study included 203 (n=83 non‐Hispanic Black [NHB] and n=120 non‐Hispanic White [NHW]) cognitively unimpaired older adults from the NYU Alzheimer's Disease Research Center. Of the 203, 115 (39NHB, 76 NHW) had tau‐PET (PI‐2620/MK‐6240) data; 195 (85 NHB, 110 NHW) had amyloid‐PET (florbetaben); 98 (39 NHB, 59 NHW) had plasma Aβ40 and Ab42 data, 93 (37 NHB, 56 NHW) had data for total tau, 135 (55 NHB, 80 NHW) had neurofilament light (NfL) and glial fibrillary protein (GFAP) data and 139 (57 NHB, 82 NHW) had pTau181 data. Plasma biomarkers assay Aβ 40, Aβ42, total tau [t‐tau], neurofilament light [NfL], glial fibrillary acid protein [GFAP], ubiquitin carboxyl‐terminal hydrolase L1 [UCH‐L1] and pTau181 were measured were measured using Quanterix SIMOA HD‐1.

**Result:**

Overall, mean (SD) age was 75.6 (6.5) NHW vs. 68.9 NHB (p=.008), BMI was 27.4 (9.7) vs. 32.4 (11.2) (p=.161), and education 17.0 (2.1) vs. 16.4 (2.32) (p=.273) for NHW vs. NHB respectively. Overall, 135 (66.5%) were females, of which 61 (73.5%) were NHB, and 62 were APOE ε4 carriers [30.5%], of which 27 were NHB. Compared to NHW, NHB subjects had significantly lower plasma Aβ40 (One‐tailed t‐test p=.006), NfL levels (p=.0005), Tau (p=.0313), pTau181 (p=.0015), and GFAP (p=.0005). NHB subjects had lower amyloid‐PET SUVR levels compared to NHW subjects (p=.0014). Baseline plasma Aβ42/40 and pTau181/Aβ42 levels were higher in NHB versus NHW participants (p=.002), consistent lower levels of amyloid pathology in NHB participants. There were no significant differences in levels of plasma Aβ42, tau‐PET SUVR, and UCH‐L1.

**Conclusion:**

In this sample of cognitively normal older adults, NHB participants had biomarker measures suggestive of lower levels of cerebral amyloid pathology. This warrants further correlation with brain imaging and cognitive outcomes, and examination of the potential impact of demographic, socio‐structural and genetic factors on observed differences. Additionally, this may imply that racial variability in amyloid threshold possibly exists before clinical AD onset, potentially lowering the proportion of NHB patients eligible for AD therapies and trials.

